# Degradation dynamics and dissipation kinetics of an imidazole fungicide (Prochloraz) in aqueous medium of varying pH

**DOI:** 10.2478/v10102-010-0047-6

**Published:** 2010-11

**Authors:** Md. Wasim Aktar, Dwaipayan Sengupta, Swarnali Purkait, Madhumita Ganguly, M. Paramasivam

**Affiliations:** 1 Pesticide Residue Laboratory, Department of Agricultural Chemicals, Bidhan Chandra Krishi Viswavidyalaya, Mohanpur-741252, Nadia, West Bengal, INDIA; 2 Department of Agricultural Chemistry and Soil Science, Institute of Agricultural Science, University of Calcutta, Kolkata, West Bengal, INDIA

**Keywords:** Prochloraz, pH, dissipation, kinetics, fungicides

## Abstract

Laboratory degradation studies were performed in water at pH 4.0, 7.0 and 9.2 using Prochloraz (450 EC) formulation at the concentration of 1.0 (T_1_) and 2.0 (T_2_) µg/mL. Water samples collected on 0 (2 h), 3, 7, 15, 30, 45, 60 and 90 days after treatments were processed for residue analysis of Prochloraz by HPLC-UV detector. In 60 days, dissipation was 89.1–90.5% at pH 4.0, 84.1–88.2% at pH 7.0, and 92.4–93.8% at pH 9.2 in both treatments. The results indicate that at pH 7.0 the degradation of Prochloraz was much slower as compared to other two. Between pH 4.0 and 9.2 the degradation of compound is little faster at pH 9.2. The half-life periods observed were 18.35 and 19.17 days at pH 4.0, 22.6 and 25.1 days at pH 7.0 and 15.8 and 16.6 days at pH 9.2 at T_1_ and T_2_ doses respectively.

## Introduction

Prochloraz (*N*-propyl-*N*-[2-(2, 4, 6-trichlorophenoxy) ethyl] -1*H*-imidazole-1-carboxamide) C_15_H_16_Cl_3_N_3_O_2_ is a broad-spectrum fungicide (Li *et al*., 2000; Vinggaard *et al*., 2006). It controls several species of *Fusarium* cause head blight; the most common in our country are *Fusarium graminearum* (Schwabe), *F. culmorum* (Smith) Sacc., *F. avenaceum* (Corda ex Fr.) Sacc. and *F. poae* (Peck) Wollenw. Another causal agent is *Microdochium nivale* (Fr.) Samuels & Hallet var. *majus* and var. *nivale,* synonym of *F. nivale* (Fr.) (Pancaldi *et al*., 1996; Balmas *et al*., 1999; Corazza *et al*., 2001). It acts by inhibiting ergosterol biosynthesis Needham & Challis, 1991). The percentage of kernels infected with *F.graminearum* and *F. culmorum* was significantly reduced, compared with the untreated kernels by 56 to 76%, with prochloraz, and by 44 to 69% with tebuconazole (Davide *et al*., 2004). Two model water pollutants, the imidazole fungicide prochloraz and the alkylphenolic compound nonylphenol diethoxylate (NP2EO), were investigated in a comparative *in vitro*/*in vivo* approach. The patterns of biochemical responses to the model pollutants were generally similar between *in vitro* and *in vivo* investigations. Levels of cytochrome P4501A (CYP1A) protein and the catalytic activity of the CYP1A-dependent enzyme 7-ethoxyresorufin-O-deethylase (EROD) were induced *in vitro* after 24 h of exposure to 1.0 microM prochloraz. *In vitro*, higher prochloraz concentrations induced only the levels of CYP1A above control levels, but not EROD activity. *In vivo* exposure of juvenile trout to 0.27 microM prochloraz resulted in an induction of CYP1A and EROD after 7 and 14 days, while 0.027 microM prochloraz had no effects (Sturm *et al*., 2001; Rivière, 1983). It has some effect with multiple modes of action on reproductive endocrine function in the fathead minnow (*Pimephales promelas*) (Ankley *et al*., 2005; 2007). No water quality standards or criteria have been established for this chemical by the U.S. or Canadian governments. Its toxicology was first evaluated by the Meeting in 1983 (Annex 1, reference 40), when an ADI of 0–0.01 mg/kg bw was established on the basis of a NOAEL of 0.9 mg/kg bw per day in a 2-year study in dogs and a NOAEL of 1.3 mg/kg bw per day in a 2-year study in rats (Kato *et al*., 1998; Laignelet *et al*., 1992). When used on crops, the residues of Prochloraz in standing water may harm beneficial flora and fauna in general and fish in particular where pisiculture is practiced in paddy crop. As there is no in formation available on this aspect of Prochloraz, it was thought imperative to investigate dissipation behaviour of this fungicide in water at different pH levels under laboratory simulated condition.

## Materials and methods

Analytical grade of Prochloraz (99.7%) was obtained from M/S Sigma-Aldrich, USA. All the solvents like dichloromethane, acetone and ethyl acetate were glass distilled before use. Sodium sulphate was washed repeatedly with distilled acetone and activated at 110 °C for 2h before use. Stock solution (100 µg/mL) was prepared in ethyl acetate and working solution was prepared by diluting it.

The pH of aqueous solution was adjusted using buffer. Buffer capsules of pH 4.0, 7.0 and 9.2 from E.Merck were used for the purpose of preparing buffer solution. One capsule was required for 100 mL of distilled water to maintain the above mentioned pH. In a series of Winchester bottles (10 L capacity) 6 L distilled water was kept and sixty capsules were added to each of the bottle. The bottles were then left in room temperature for overnight to homogenize the buffer solutions. For carrying out laboratory experiment, water (6L) of each pH triplicate was spiked at 1.0 (T_1_) and 2.0 (T_2_) µg/mL with Prochloraz formulation and was stored in Winchester bottles from October to February 2006*–*07 under room temperature (15*–*31.5 °C). Untreated control was also carried out simultaneously. Samples were drawn periodically on 0 (2h), 3, 7, 15, 30, 45, 60, and 90 days after treatments and analyzed for Prochloraz residues.

Representative 200 mL water sample was taken in 1 L separating funnel and 5*–*10 g sodium chloride was added to it. It was extracted thrice (100, 50, 50 mL) with dichloromethane by liquid-liquid partitioning. Organic phases were combined, passed through anhydrous sodium sulphate and concentrated on a rotary vacuum evaporator under reduced pressure at 40 °C followed by a gas manifold evaporator till near dryness. Final solution was made to 5 mL in acetonitrile and subjected to HPLC analysis. No clean-up was required as no interference peaks were observed during analysis.

The residues of Prochloraz were analyzed on HPLC (Agilent Technologies 1200 Series) equipped with variable wavelength detector (VWD) using column (Shandon Hypersil 250 X 4.6 mm ODS 5 (RPC_18_). The wavelength (λ _max_), mobile phase and flow rate was 212 nm, methanol-water (85:15, v/v) and 1mL/min, respectively. The retention time, limit of detection (LOD) and limit of quantification (LOQ) were 5.13 min, 0.01 µg/g and 0.05 µg/g, respectively.

## Results and discussion

Average recoveries of Prochloraz from water fortified at 0.25 and 1 µg/mL varied from 90*–*96% at pH 4.0, 88*–*98% at pH 7.0 and 88*–*97% at pH 9.2.

Residues of Prochloraz in water at different pH levels are presented in Table 1. As evident from the data, at pH 4.0, initial residues of 0.95 µg/mL in treatment T_1_ dissipated to 0.85 µg/mL in 3 days, 0.42 and 0.09µg/mL in 30 and 60 days, respectively. Corresponding dissipation were 10.5, 55.8 and 90.5%, respectively. In treatment T_2_, initial residues of 1.93 µg/mL dissipated to 1.64, 0.69 and 0.21 µg/mL in 3, 30 and 60 days after application with corresponding dissipation of 15.0, 64.3 and 89.1%, respectively.

**Table 1:** Dissipation of Prochloraz residues in water at different pH.

	Residues (µg/mL) ± SD (% of Dissipation)					
	
	pH 4.0		pH 7.0		pH 9.2	
			
Time (in days)	1.0 µg/mL	2.0 µg/mL	1.0 µg/mL	2.0 µg/mL	1.0 µg/mL	2.0 µg/mL
0	0.95±0.09	1.93±0.01	0.93±0.03	1.95±0.07	0.97±0.06	1.96±0.02
	(–)	(–)	(–)	(–)	(–)	(–)
3	0.85±0.03	1.64±0.03	0.88±0.06	1.68±0.01	0.73±0.09	1.54±0.11
	(10.5)	(15.0)	(5.4)	(13.9)	(24.7)	(21.4)
7	0.73±0.05	1.47±0.09	0.76±0.13	1.51±0.02	0.62±0.01	1.39±0.04
	(23.2)	(23.8)	(18.3)	(22.6)	(36.1)	(29.1)
15	0.62±0.01	1.24±0.01	0.64±0.04	1.27±0.05	0.45±0.08	1.13±0.03
	(34.7)	(35.8)	(31.2)	(34.9)	(53.6)	(42.4)
30	0.42±0.04	0.69±0.06	0.45±0.08	0.79±0.01	0.28±0.02	0.59±0.06
	(55.8)	(64.3)	(51.6)	(59.5)	(71.1)	(69.9)
45	0.19±0.02	0.39±0.03	0.27±0.06	0.48±0.04	0.13±0.03	0.29±0.02
	(80.0)	(78.0)	(71.0)	(75.4)	(86.6)	(85.2)
60	0.09±0.05	0.21±0.02	0.11±0.04	0.31±0.07	0.06±0.09	0.15±0.05
	(90.5)	(89.1)	(88.2)	(84.1)	(93.8)	(92.4)
90	BDL	BDL	0.07±0.07	0.17±0.02	BDL	BDL
			(92.5)	(91.3)		

BDL=Below Detectable Limit

At pH 7.0, initial residues of 0.93 µg/mL in T_1_ treatment, dissipated to 0.88, 0.45, 0.11 and 0.07 µg/mL in 3, 30, 60 and 90 days after treatment with corresponding dissipation of 5.38, 51.61, 88.17 and 92.5% respectively, whereas in T_2_ initial residues of 1.95 µg/mL dissipated to 1.68, 0.79, 0.31 and 0.17 µg/mL in 3, 30, 60 and 90 days after treatment showing dissipation of 13.85, 59.49, 84.10 and 91.3%, respectively.

At pH 9.2, from treatment T_1_ initial residues of 0.97 µg/mL dissipated to 0.73, 0.28 and 0.06 µg/mL in 3, 30 and 60 days after treatment with corresponding dissipation of 24.74, 71.13 and 93.8%, respectively. In T_2_ residues of 1.96 µg/mL on 0 (2 h) day dissipated to 1.54, 0.59 and 0.15 in 3, 30 and 60 days after treatment and corresponding dissipation was 21.4, 69.9 and 92.4%, respectively.

It is clear that Prochloraz residues in water dissipated more than 80% in 60 days at pH levels of 4.0, 7.0 and 9.2 in both treatments under laboratory conditions under temperature ranging from 15 to 31.5°C. The degradation was slow during first 3 days followed by relatively faster degradation from 3^rd^ day onward to 45 days after which it became slow.

Almost identical degradation ranging from 89.1 to 93.8% at two pH levels (viz. pH 4.0 and 9.2) during 60 days study indicate that there was no significant effect of these two pH on degradation, although dissipation was a little faster at pH 9.2 throughout the studies. More than 91% degradation at 90 days after treatment indicates that degradation was much slower at pH 7.0 than that of other two. Half-life values at pH levels of 4.0, 7.0 and 9.2 varied from 18.4 to 19.2, 22.6 to 25.1 and 15.8 to 16.6 days, respectively (Table 2). At all pH levels degradation was observed to be faster in T_1_ than T_2_. The slow dissipation at higher rate could attribute to inhibition of microbial activity.

**Table 2:** Regression equation, Correlation Co-efficient and half-life for the dissipation of Prochloraz in water at different pH.

pH	Treatments (µg/mL)	Regression Equation	Correlation co-efficient	Half-life (days)
4.0	1.0	y=2.007–0.0164x	0.977	18.35
	2.0	y=2.29–0.0157x	0.995	19.17
7.0	1.0	y=1.9863–0.0133x	0.975	22.64
	2.0	y=2.2641–0.0121x	0.992	25.08
9.2	1.0	y=1.953–0.019x	0.992	15.84
	2.0	y=2.2824–0.0181x	0.995	16.63

Similar observations have been reported in degradation of fenoxanil in water where dissipation was independent of pH and was subject to photodegradation following pseudo first order kinetics. Dissipation of Nonylphenol Diethoxylate residues has also been reported following pseudo first order kinetics in river waters (Cravedi *et al*., 2001). Whereas in another studies, decomposition of Nonylphenol Ethoxylates and bixafen in water as pH and dose dependent, resulted in faster dissipation under alkaline conditions and at low dose of application (Debrauwer *et al*., 2001; Gac *et al*., 2001). Slightly faster dissipation of Prochloraz at alkaline pH has been observed in our studies also.

## Conclusions

Considering rapid dissipation of Prochloraz at the tested doses in water system, its much faster degradation can be expected under field condition. Consumption of vegetables from Prochloraz treated field could not be harmful for health as no residues of the herbicide would be found in the harvested plant samples as the dissipation of Prochloraz would be higher in plant system due to higher enzymatic activity. Hence, the tested doses can be considered safe from the point of view of health hazards, environmental pollution and ground water contamination due to its residual effects.
